# Production of Hybrids between Western Gray Wolves and Western Coyotes

**DOI:** 10.1371/journal.pone.0088861

**Published:** 2014-02-25

**Authors:** L. David Mech, Bruce W. Christensen, Cheryl S. Asa, Margaret Callahan, Julie K. Young

**Affiliations:** 1 U. S. Geological Survey, Northern Prairie Wildlife Research Center, Jamestown, North Dakota, United States of America; 2 Department of Population Health and Reproduction, School of Veterinary Medicine, University of California Davis, Davis, California, United States of America; 3 Research Department, Saint Louis Zoo, St. Louis, Missouri, United States of America; 4 Wildlife Science Center, Forest Lake, Minnesota, United States of America; 5 U. S. Department of Agriculture, Wildlife Services, National Wildlife Research Center, Department of Wildland Resources, Utah State University, Logan, Utah, United States of America; Virginia Tech Virginia, United States of America

## Abstract

Using artificial insemination we attempted to produce hybrids between captive, male, western, gray wolves (*Canis lupus*) and female, western coyotes (*Canis latrans*) to determine whether their gametes would be compatible and the coyotes could produce and nurture offspring. The results contribute new information to an ongoing controversy over whether the eastern wolf (*Canis lycaon*) is a valid unique species that could be subject to the U. S. Endangered Species Act. Attempts with transcervically deposited wolf semen into nine coyotes over two breeding seasons yielded three coyote pregnancies. One coyote ate her pups, another produced a resorbed fetus and a dead fetus by C-section, and the third produced seven hybrids, six of which survived. These results show that, although it might be unlikely for male western wolves to successfully produce offspring with female western coyotes under natural conditions, western-gray-wolf sperm are compatible with western-coyote ova and that at least one coyote could produce and nurture hybrid offspring. This finding in turn demonstrates that gamete incompatibility would not have prevented western, gray wolves from inseminating western coyotes and thus producing hybrids with coyote mtDNA, a claim that counters the view that the eastern wolf is a separate species. However, some of the difficulties experienced by the other inseminated coyotes tend to temper that finding and suggest that more experimentation is needed, including determining the behavioral and physical compatibility of western gray wolves copulating with western coyotes. Thus although our study adds new information to the controversy, it does not settle it. Further study is needed to determine whether the putative *Canis lycaon* is indeed a unique species.

## Introduction

Two schools of thought dominate the molecular-genetics literature on *Canis* sp. (wolves) in the Great Lakes region of the U. S. and Canada: they are hybrids between *Canis lupus* L. (gray wolf) and *Canis latrans* Say (coyote) [1], or they are hybrids between the gray wolf and *Canis lycaon* Schreber (the eastern wolf), a putative unique species [2]. Researchers have presented molecular-genetic evidence for a new interpretation of the taxonomy of North American *Canis* species. They have concluded that the gray-wolf subspecies *Canis lupus lycaon*, is a separate species, similar to *Canis rufus* Audubon and Bachman (red wolf), that should be named *Canis lycaon* Schreber, the eastern wolf [2]. The study was based on analyses of 8 microsatellite loci and mitochondrial DNA (mtDNA) control-region sequences from wolves of southeastern Ontario from the 1960s. None showed gray wolf mtDNA, and estimates were that mtDNA sequences from both the eastern wolf and red wolf diverged 150,000–300,000 years ago from coyotes as compared to a divergence from the gray wolf of around 2 million years. Based on this evidence the researchers suggested that both the red wolf and eastern wolf evolved in North America along with the coyote, as opposed to the gray wolf which evolved in the Old World. These workers [2] also compared their microsatellite allele frequencies with published frequencies of wolves and coyotes from other areas [3], [4] and found both microsatellite evidence and mtDNA evidence of eastern wolves as far west as Manitoba.

Previously others [1], [5] had considered the same mtDNA haplotypes as evidence of gray wolf x coyote hybridization, and that school continued to interpret them that way [6], [7]. The challengers [2] based their interpretation on the fact that those haplotypes have not been found in extant non-hybridizing coyote populations, and those whose work was challenged [8] agreed that the new interpretation [2] should be further tested. Several other authors accepted the eastern wolf as a species [9]–[16]. However, workers [17] using 48,000 single-nucleotide polymorphisms (SNPs) rejected that interpretation and favored the wolf x coyote interpretation, although others [18], [19] disagreed.

Regarding the current wolf population in the Great Lakes region, from northeastern Ontario west across Ontario, Michigan, Wisconsin, Minnesota, and at least part of Manitoba, the disparate schools of thought are that those wolves are either hybrids between gray wolf x eastern wolf [14], [20] or gray wolf x coyote [1], [7]. The gray wolf has been on the U.S. Endangered Species List since 1967 and protected by the Endangered Species Act of 1973, was delisted in 2012, but that delisting is being legally contested. In addition, claims have been made based on genetic analyses that the native Great Lakes' wolves were not restored [6] but *cf* [15], [21] and that the Great Lakes' wolves are a “unique population or ecotype of gray wolves” [7] but *cf* [22]. The U.S. Fish and Wildlife Service has accepted the interpretation that the eastern wolf is a valid new species [18] that might be considered endangered [23]. Therefore it is important to try to determine the correct interpretation of the coyote-like mtDNA haplotypes in gray wolves by examining evidence other than genetic data.

The crux of the controversy rests on the question as to whether male gray wolves can hybridize with female coyotes. Where definitively-identified gray wolves and coyotes are sympatric (western North America) there is no documented hybridization between the two, and wolves kill coyotes [24]. Furthermore, where the putative eastern wolf and eastern coyotes are sympatric (eastern North America) they do hybridize [25], [26].

Experimental hybridization between known gray wolves and western coyotes has not been attempted, but a male eastern coyote and putative eastern wolf did hybridize in captivity [25]. However, for coyote mtDNA to appear in a wolf, a female coyote and male wolf would have to mate. Such a mating could present significant behavioral, physical, and possibly physiological impediments. Because there is no documentation that gray-wolf semen can fertilize western coyote ova, we used artificial insemination to test this possibility.

## Methods

Eight male, western gray wolves from a den in northern British Columbia used for semen collection were housed together along with a proestrous female in a 0.3- hectare enclosure at the Wildlife Science Center in Columbus, MN and fed road-killed white-tailed deer (*Odocoileus virginianus*) ad libitum. They were vaccinated annually with Duramane Max 5 and Rabvac (Boehringer Ingelheim). We collected semen either manually or by electroejaculation (EE) on 16 and 18 January 2012 and on 24 and 25 January 2013 (Table 1).

**Table 1:** Captive western, gray wolf semen analysis results at time of collection.

Collection Date	Wolf ID (Date of birth)	Collection Method	Percent motile sperm	Sperm Vigor*	Volume (ml)	Comments
16 Jan 2012	486	M	90	4	4.5	
	(19 May 2011)					
	487	M	50	4	4	
	(19 May 2011)					
18 Jan 2012	487	M	60	3	0.6	
	494	EE	75	4	5.3	Some sperm
	(19 May 2011)					agglutination
24 Jan 2013	480	EE	75	3–4	4.4	Some blood
	(26 Apr 2008)					contamination
	486	EE	75	4–5	13	
	487	EE	80	4	4.5	Some sperm
						agglutination
	494	EE	75	4	5	
25 Jan 2013	497	EE	60	4	4	Slight blood
	(8 Apr 2010)					contamination
	499	EE	50	1	3	Urine
	(29 Apr 2011)					contamination
	500	EE	60	4	4	Sperm
	(29 Apr 2011)					agglutination
	501	EE	30	4	5	Urine
	(29 Apr 2011)					contamination

*Vigor: 1 = slight side-to-side motion, no forward progress; 3 = rapid side-to-side, forward progress in spurts; 4 = slow, steady, forward progress; 5 = rapid, steady, forward progress.

### Manual Semen Collection

The handler stimulated the penis of the wolf via manual stroking through the prepuce. When engorgement of the bulbous glandis was detected, the prepuce was retracted proximal to the bulbous glandis and the penis grasped proximal to the bulbous glandis between the thumb and fingers of one hand. The tip of the glans penis was directed into a glass funnel with a plastic 15-cc centrifuge tube attached to the base and secured inside a 50-ml tube as a warm-water bath at approximately 37°C to prevent cold-shock to the sperm.

### Electroejaculation

We collected semen from the wolves under general anesthesia (6.25 mg/kg IM ketamine hydrochloride: Ketaset, Boehringer Ingelheim Vetmedica Inc., St. Joseph, MO, USA, and xylazine: Rompun, Bayer Corp., Shawnee Mission, KS, USA) and maintained by supplemental ketamine. Just prior to stimulation we flushed the urinary bladder with sterile saline to minimize urine contamination of samples. We passed an 8-French polypropylene catheter (Sovereign, Tyco Kendall Healthcare, Mansfield, MA, USA) coated with sterile lubrication through the urethra into the bladder. After urine was aspirated, we repeatedly infused saline and aspirated until the effluent was clear.

We collected semen by EE (Model 12 electroejaculator: G & S Instruments, Midlothian, TX, USA) using a 3-cm-diameter probe with three, 7.5-cm long, linear electrodes (PT Electronics, Boring, OR, USA) placed ventrally in the rectum above the prostate gland and pudendal nerve. We increased the stimulation slowly until the hind-limbs extended, returned to zero, and repeated rhythmically at gradually increasing voltage, with a cycle of approximately 5 seconds, until ejaculation. Maximum current did not exceed 300 mAmp, the measure of current reaching the tissues. We repeated stimulation as long as the sample appeared cloudy, an indicator of sperm presence.

### Semen Analysis and Handling

Immediately following collection, we separated a drop of semen for analysis. We estimated percent motility and status using a phase-contrast microscope at 200X and calculated concentration using a Makler Counting Chamber (Sefi-Medical Instruments, Haifa, Israel). We spun the remaining sample at 700×g for 10 minutes, discarded the supernatant (seminal fluid), and resuspended the sperm pellet in a room-temperature, Chill-5 extender (Minitube of America, Verona, WI 53593) developed to preserve domestic dog semen for overnight shipment. On the day of collection we packaged the sample plus Chill 5 in a Chill-5 Shipping Kit (Minitube of America), an insulated container with ice packs placed so that the sperm cool at a controlled rate during transport, and shipped them overnight to the USDA National Wildlife Research Center's Predator Research Facility in Millville, UT, USA. There they were to be used to inseminate western coyotes in the captive colony whose ancestors were wild-caught in such western states as Idaho, Utah, Wyoming, and Montana.

### 2012 female coyote estrus synchronization and artificial insemination

Six female coyotes located at the Predator Research Facility, used in 2012, ranged from 3–5 years old (3.8±0.4). We placed deslorelin implants (2.1 mg, Ovuplant®, Peptech Animal Health Pty Limited, NSW, Australia) subcutaneously in the inguinal region of the six females on 3 January, 2012. Six adult female coyotes that were 1-7-years-old (3.7+0.9) and housed at the same facility did not receive implants and were monitored as controls (Table 2). We measured serum progesterone concentrations by chemiluminescent microparticle immunoassay on 16 January (treated animals) and 19 January (both treated and control animals). Four treated coyotes with rising values above baseline (>2 ng/ml) were selected for insemination (two 3-year olds, one 4-year old, and one 5-year old).

**Table 2:** Serum progesterone concentrations from captive, western, coyote females in 2012.

Coyote	January 16 Serum Progesterone concentration (ng/ml)	January 19 Serum Progesterone concentration (ng/ml)
B^*^	3.9	8.8
Peg^*^	3.8	6.0
Snippy^*^	5.0	21.1
Shadow^*^	11.0	40.5
<$>\raster(70%)="rg1"<$>		
A	20.3	15.7
Chili	0.9	1.0
Bee		1.2
F		3.8
Sub		0.4
Inga		0.5
E		0.3
Zig		1.4

Animals in bold were treated with an Ovuplant subcutaneous implant on 3 January. The others were used as controls. Four coyotes (*) were artificially inseminated on 17 January (60% progressively motile sperm) and 19 January (30% progressively motile sperm). One coyote (<$>\raster(70%)="rg1"<$>) was confirmed pregnant with a single fetus and later judged to have resorbed one.

We performed inseminations on 17 and 19 January 2012 after physically restraining coyotes, anesthetizing them with xylazine (1.25 mg/kg IV) and ketamine (6.25 mg/kg IV), and placing them in sternal recumbency with the hind legs in flexion.

We inserted a cotton swab into the vagina, turned and removed the swab, and then rolled it on a glass slide subsequently stained with a modified Wright Giemsa stain protocol for vaginal cytological evaluation. We removed the subcutaneous, deslorelin implant via a small incision in the skin. We visualized the cervix by passing a rigid uretero-renoscope (43 cm×9.5 French; Karl Storz Veterinary Endoscopy-America, Goleta, CA) through the vaginal canal. We then advanced a 5-French, flexible pipette through the endoscope and through the external os of the cervix for 5 cm. We injected 3–5 ml of extended, gray-wolf semen (concentration unknown) through the pipette slowly into the uterus before withdrawing the pipette and endoscope. We kept the hind end of the female slightly elevated for 20 minutes during the recovery from anesthesia to prevent retrograde flow of the semen. We used a commercial relaxin assay [27], [28] to diagnose pregnancy at approximately 30-days gestation (16 February 2012). We took radiographs at approximately 56-days gestation (15 March 2012) to count fetuses.

### 2013 female coyote estrus synchronization and artificial insemination

We implanted 10 female coyotes aged 3–6 years (mean age, 4.28 years ±0.3 SE) with a subcutaneous, deslorelin implant (2.1 mg, Ovuplant®) in the inguinal region on January 10, 2013 (Table 3). Serum progesterone concentrations were evaluated by chemiluminescent microparticle immunoassay on 21, 23, and 25 January. We used a rising trend in serum progesterone above baseline (>2 ng/ml) as a guide for deciding when to inseminate and selected five coyotes (three 4-year olds, one 5-year old, and one 6-year old) for insemination. We inseminated one female on 22 January 2013, three on 25 January 2013, and two on 26 January 2013, once each, except one female inseminated on 22 January and 25 January. We tested for pregnancy by serum-relaxin assay on 6 March, 2013, and radiographically evaluated the females on 19 March 2013 to count fetuses. We performed implant removal and insemination as described for 2012.

**Table 3:** Serum progesterone concentrations from captive, western, coyote females in 2013.

Coyote	January 21 Serum Progesterone concentration (ng/ml)	January 23 Serum Progesterone concentration (ng/ml)	January 25 Serum Progesterone concentration (ng/ml)
A	2.1	1.5	1.9
B*<$>\raster(70%)="rg1"<$>	12.5	29.5	37.8
C	6.6	2.9	3.8
D*	5.9	3.9	17.5
E*	3.5	5	2.6
F	3	2.4	3.5
G	4.1	3.6	3.9
H	1.1	0.7	0.4
I*<$>\raster(70%)="rg1"<$>	10.8	32.8	60.7
J*	1.6	2.5	4.2

All animals were treated with an Ovuplant subcutaneous implant on 10 January. Five coyotes (*) were artificially inseminated on 22 January (Coyote B; 30% progressively motile sperm, low dose), 25 January (Coyotes B, E, and I; 80% progressively motile sperm, adequate dose), and 26 January (Coyotes D and J; 10% progressively motile sperm). Two coyotes (<$>\raster(70%)="rg1"<$>) were confirmed pregnant with 6–8 fetuses, each.

### Observing Females during Parturition

We maintained all pregnant females individually in clover pens (0.1 ha) with an infrared camera in den boxes to monitor them for signs of labor without disturbing them starting at 60 days estimated gestation. During the day, the pens were within view of areas normally used by staff, and we used binoculars and spotting scopes to assess their status. At night, staff checked on the females at hourly intervals in 2012 and at 3-hour intervals in 2013 via the video feed from the infrared camera or by using night vision binoculars.

### Ethics Statement

This study was carried out in strict accordance with the recommendations in the Guide for the Care and Use of Laboratory Animals of the National Institutes of Health. The protocol (QA-1953 and Amendment 1) was approved by the IACUC of the U. S. Department of Agriculture, Animal and Plant Health Inspection Service, Wildlife Service National Wildlife Research Center. Insertion of deslorelin implants, artificial insemination, and C-section were performed under ketamine and xylazine anesthesia, and all efforts were made to minimize pain or discomfort.

### Statistics

Hypothesizing that the deslorin-implanted females would show elevated progesterone levels, we log-transformed serum-progesterone concentrations and compared the means of the treated sample with those of the controls by two-sample t-test. We also compared proportion of coyotes with elevated progesterone (>2.0 ng/ml) in the treated sample with that proportion in the control sample by one-tailed Fisher's exact test.

## Results

We obtained motile sperm from three wolves in 2012 and eight in 2013, but the quality varied among males based on assessment of side-to-side motion and degree and speed of forward progress (Table 1). Two samples contained some blood contamination, perhaps due to irritation of the bladder or urethra by the catheter; two were contaminated by urine; and two contained sperm that were agglutinating.

In 2012, serum progesterone concentrations were elevated above baseline (>2.0 ng/ml; range of 1.0 to 40.5 ng/ml, median  = 12.3 ng/ml) in 5 of 6 treated females compared with an elevation in 1 of 6 females in the control group (range of 0.3 to 3.8 ng/ml, median  = 0.9 ng/ml) (Table 2). Coyotes receiving implants had higher serum progesterone concentrations than control coyotes (P = 0.016) and treated animals had elevated serum progesterone levels over baseline compared to control animals (P = 0.04). Vaginal cytological evaluation in the treated females both in 2012 and 2013 revealed cells consistent with estrogen influence.

In 2012 relaxin assays indicated that 5-year-old, nulliparous female (Shadow) was pregnant. At approximately 56 days gestation we moved her to a smaller pen to facilitate monitoring, ultrasound, and radiography and found she had a single fetus. She did not use her den box after the move, so we relied on night-vision binoculars exclusively to observe her. She went into labor after 63 days gestation (22 March 2012). After she underwent 2–3 hours of labor without delivery, we transported her to a veterinary hospital for an emergency c-section. The fetus did not survive and was estimated to have been dead for several hours. There was also sign of a resorbed fetus. The female recovered without further incident.

In 2013 relaxin assays indicated that two females became pregnant.

Nulliparous, 6-year-old Female B was estimated to have at least 6 fetuses. She used her den box and delivered at least 7 pups on day 60 (23 March 2013). Via video monitoring we saw that the first pup whelped was stillborn. When Female B left the den for food, we saw that six pups were alive, although one appeared weak, and the stillborn pup was missing. This female cared for and nursed six pups for 13 days before we removed them for hand rearing.

Four-year-old Female I had given birth to 7 coyote pups in 2011 and 8 in 2012. In 2013, she had at least 8 hybrid fetuses. She did not use her den box. We observed through a spotting scope Female I going into labor during dusk the evening of day 61, and she delivered four pups before it was too dark to observe (24 March 2013). The first two pups born were moving, and the female placed them in separate ground divots. The next pup may have been stillborn. We never saw it move, and the female carried it around to multiple divots. Before delivery of the fourth pup, she ate at least two of the three pups including the third-one born. We confirmed that the fourth pup was alive based on pup sounds. At that time it was too dark to monitor, even with night-vision binoculars, without disturbing the female. By morning there were no pups.

**Figure 1:**
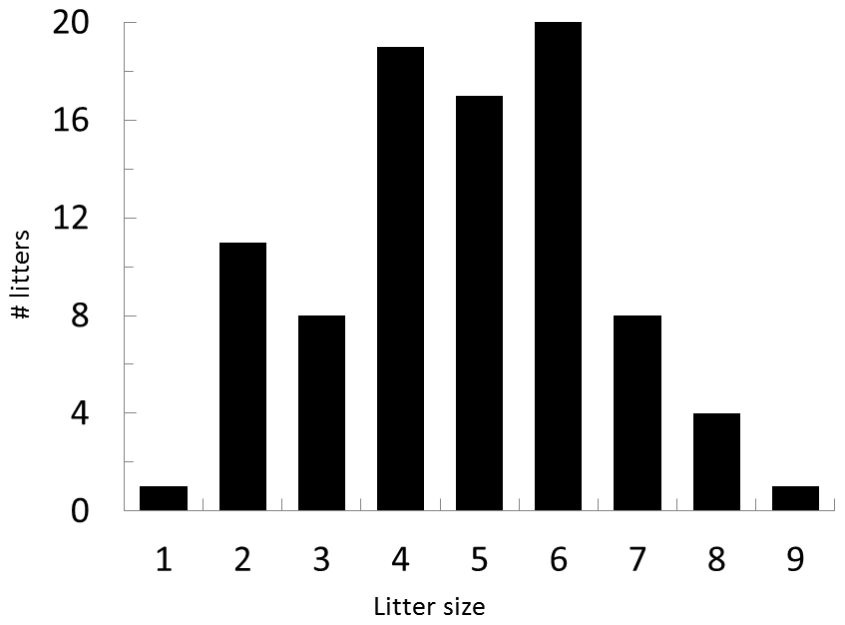
Number of litters and litter sizes of western, coyote pups produced at the U S. Department of Agriculture Wildlife Services National Wildlife Research Center Predator Research Facility between 2005–2012.

Based on 2-day litter counts of all pups born to coyotes at the UT facility from 2005 to 2012 (n = 89), the frequency of singleton births is low (0.01) while the frequency of producing 6 pups is high (Figure 1). Eating pups had been extremely rare to non-existent at this facility and did not occur with either of the two previous litters of Female I that ate her pups in 2013.

**Figure 2:**
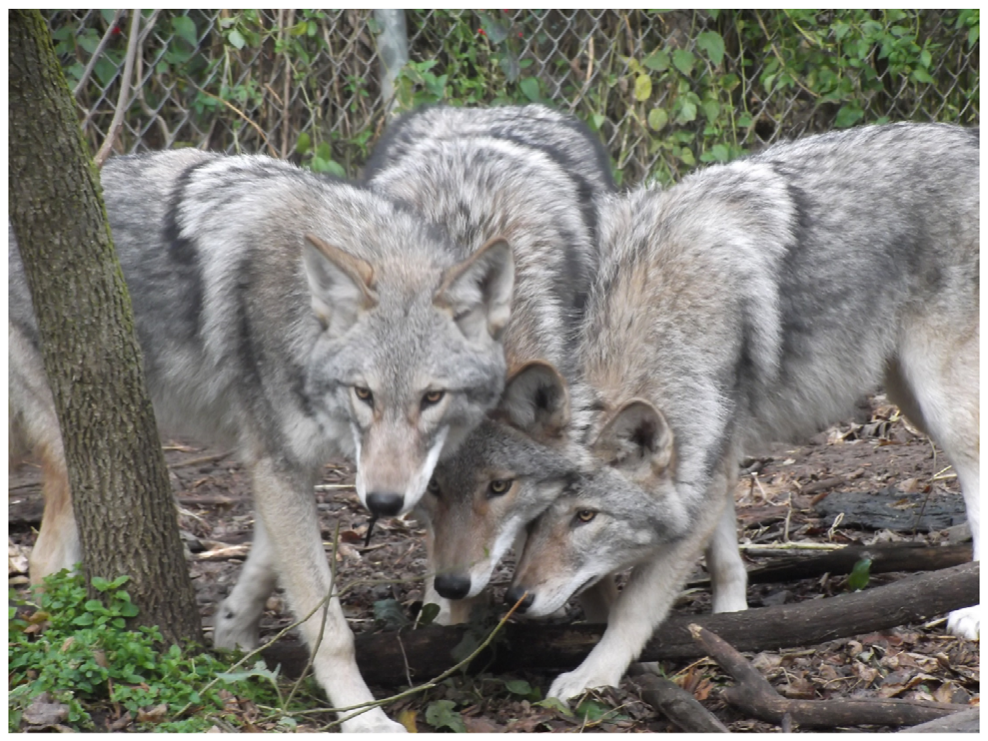
Three 6–7-month-old, littermate, F_1_ hybrids between a male, western, gray wolf and a female, western coyote resulting from artificial insemination.

**Figure 3:**
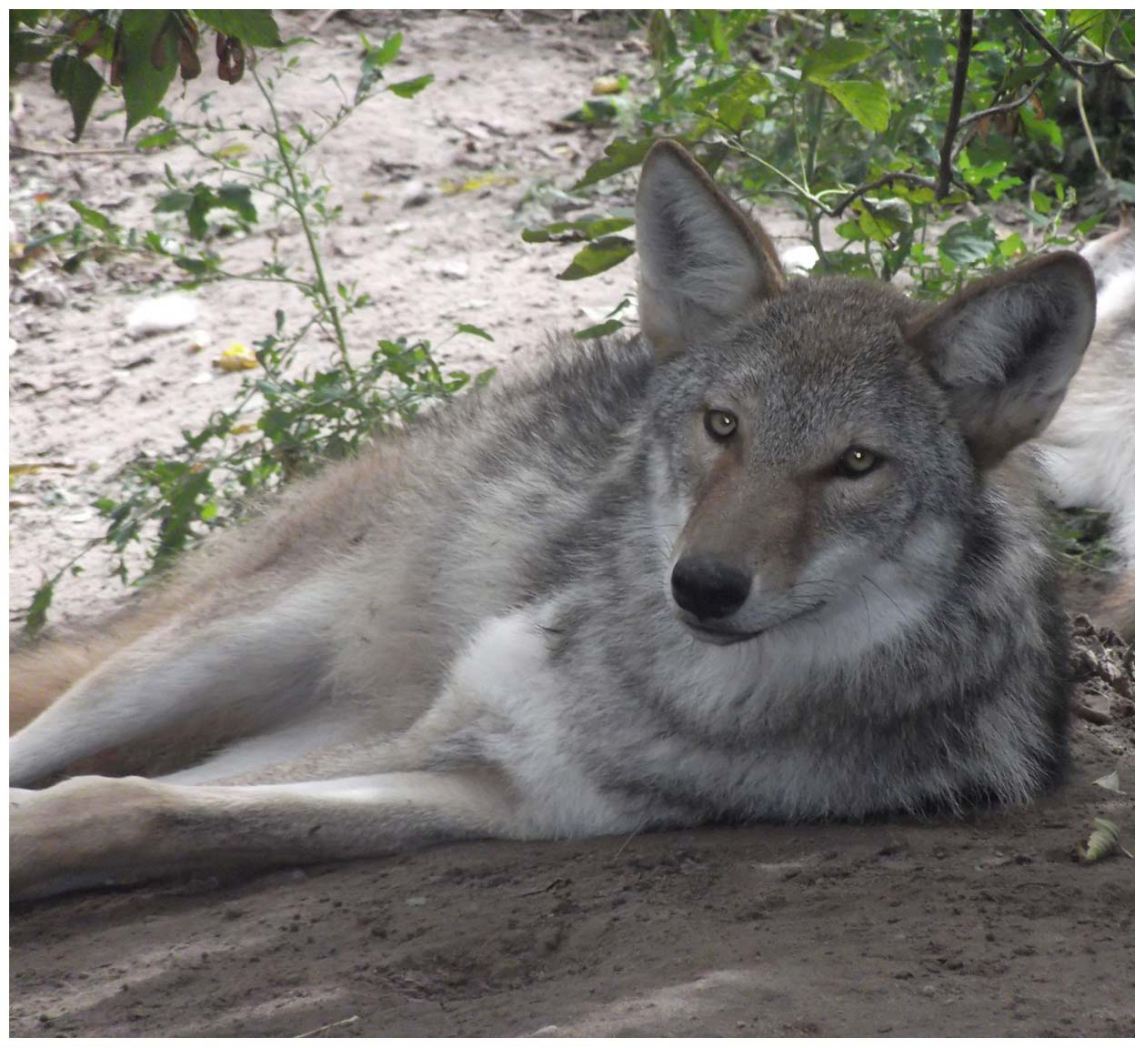
Facial view of two 6–7-month-old F_1_ hybrids between a male, western, gray wolf and a female, western coyote resulting from artificial insemination.

**Figure 4:**
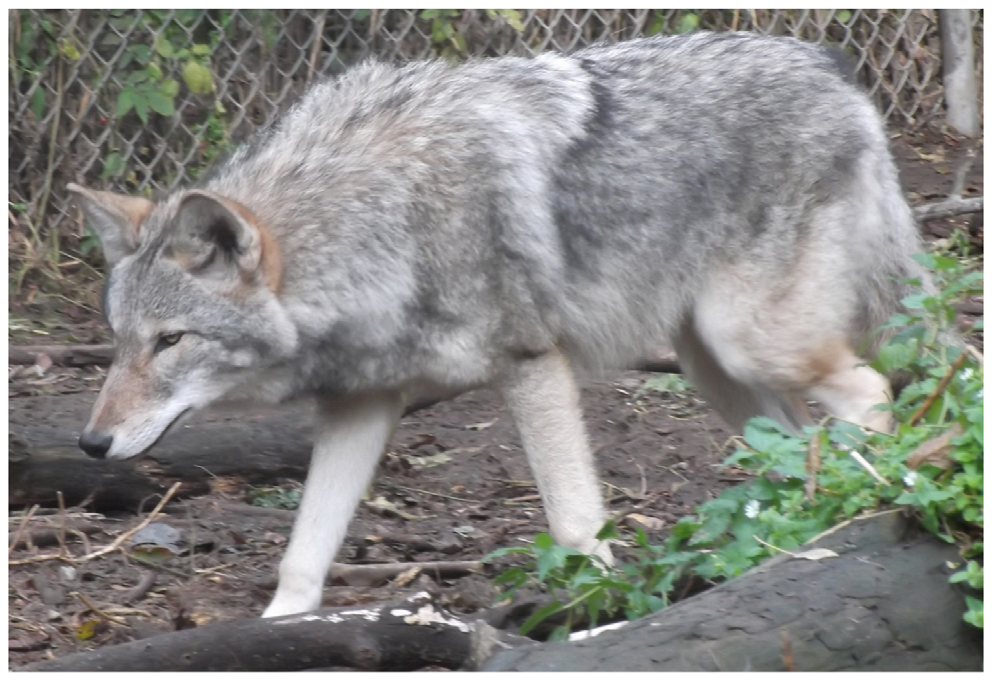
Side view of a 6–7-month-old F_1_ hybrid between a male, western, gray wolf and a female, western coyote resulting from artificial insemination.

We transported the 6 surviving pups when 13-days old to the Wildlife Science Center, Columbus, Minnesota for rearing by humans, and as of this writing (January 2014) all were surviving and were healthy. By October the hybrid pups had characteristics of both gray wolves and coyotes (Figures 2, 3, 4). As of 6 November 2013, the two female hybrids weighed 17.6 and 21.8 kg, and the four males varied between 20.1 and 26.0 kg. For comparison, 10 western female coyotes 1-7-years old from our colony weighed 10.1–16.2 kg about the same time (mean  = 12.4±0.56 kg SE), and 20 males 2–7-years old weighed 10.1–17.1 kg (mean  = 14.4±0.41 kg SE). Thus the hybrid 6-7-month old males weighed significantly more than the adult male coyotes (P>0.0001) and overlapped in weight with the range of weights of male wolf pups from south-central Alaska (mean  = 26.2±2.3 kg SE) [29]. Because we only had two female hybrids we did not test the significance of the weight difference with coyotes, but the same trend is apparent.

## Discussion

A logical hierarchy of questions seeking to determine whether free-ranging western coyotes and western gray wolves might have hybridized in the past would include the following:

1. Do female western coyotes mate with male western wolves and produce fertile offspring?
2. Will female western coyotes mate with male western wolves and produce fertile offspring?
3. Can female western coyotes mate with male western wolves?
4. If they mate, can they produce fertile offspring?
5. Physiologically, behaviorally, and physically can female western coyotes mating with western wolves produce fertile offspring?
6. Physiologically, behaviorally, and physically can female western coyotes impregnated with western wolf semen produce offspring?

To date there is no information to answer questions 1–5, and there is evidence against 1 and 2 [24]. Our results show that western gray-wolf semen can inseminate at least some western coyotes and that those coyotes can deliver viable pups. However, our overall results were equivocal enough to make it worthwhile to consider the extent to which the failures likely resulted from our methods versus from some inherent problems in compatibility between western gray wolf semen and western coyote production of pups resulting from that insemination.

We used semen from eight individual gray wolves and attempted to inseminate nine captive western coyotes over two breeding seasons, which resulted in three coyote pregnancies. Of those, one had trouble delivering a single pup and had to be given a C-section, but the fetus had died. There was also evidence of a resorbed fetus. A second female produced at least seven pups, the first of which was stillborn, but six survived. The third female produced at least 8 pups but consumed them all within 24 hours.

A summary of the various failures at least some coyotes experienced between attempted insemination and normal pup delivery and care follow: (1) lack of conception or early embryonic death, (2) low litter size, (3) resorbed fetus, (4) inability to deliver, (5) stillborn fetus, and (6) consumption of pups. The coyote that ate her pups showed more difficulty than normal in delivery, behaving as though she was in severe pain.

The following possible methodological problems could have caused these failures: (1) inadequate quantity or quality of gray-wolf semen, (2) faulty timing of the insemination, and (3) artificial conditions of captivity. On the other hand, we cannot rule out reproductive incompatibility for at least some of the failures. Based on our experience with assessing sperm quality and artificially inseminating domestic dogs, we can only judge that most of the failures to conceive were probably caused by poor semen quality or quantity or by mistiming of inseminations. Some 68–78% of domestic dogs induced with Ovuplant in late anestrus became pregnant [30], [31].

In both years, semen was of generally poorer quality (percent motility and normal morphology) than is typical later in the wolf breeding season (C. S. Asa et al. unpublished). Female wolves at the Minnesota facility are receptive to mating during the first week in February through early March (earliest mating: 1 February; mean: 16 February) [32]. Males only produce sperm around the breeding season, with the highest quality observed in the middle of that period [33]. In contrast, coyotes at the Utah facility mate as early as mid-January, two-six weeks earlier than the wolves in our study (J. K. Young, unpublished). Because we needed to induce estrus and ovulation in the female coyotes to time the inseminations, it was important to stimulate them very early in their breeding season to minimize the chances that they would ovulate naturally before we could inseminate them. Thus at the time of those earlier ovulation dates, our wolves were not yet producing high-quality sperm.

However, some of our other findings could more likely have been a result of interspecies' reproductive incompatibility: resorption of a fetus, production of only a single pup, death of that pup, and difficult delivery that could have led to pup consumption. The difficult delivery in 2012 leading to the need for a C-section probably resulted from the fact that the fetus was extra large, a condition common in domestic dogs bearing a single pup. The hybrid fetus weighed 271 gm, 29% larger than a typical coyote pup at birth (Table 4). The successful delivery in 2013 included six pups whose average weights at 2 weeks were similar to the average weight of similarly-aged coyote pups from similarly-sized litters (Table 5). It is of interest, however, that one of the 2-week-old hybrids at 720 gm was 8% heavier than the largest pup of five litters of six coyotes each at the same age. The SE of the mean weight (630.7 gm) of the hybrid litter (31.1) was 9% greater than the highest SE of each of the five coyote litters (Table 5). This greater variation in hybrid pup size, if typical, might cause delivery problems in some coyotes.

**Table 4:** Size of a single hybrid fetus at C-section compared with a typical western coyote pup at birth [34].

Measurement	Coyote at birth	Hybrid*
Weight	210 g	271 g (2.5–3 days)
Total length	27 cm	31.4 cm (4 days)
(tip of nose to tip of tail)		
Body length	20 cm	24.5 cm (7 days)
(total length – tail length)		
Head length	5.9 cm	6.6 cm (4 days)
(tip of nose to crest of head behind ears)		
Hind foot length	4 cm	4.1 cm (0 days)
(longest toenail to heel)		
Head circumference	NA	12.4 cm
(circumference directly in front of ears)		
Shoulder circumference	NA	13 cm
(circumference taken around shoulders)		

*(Ages) are those that a typical coyote pup would have attained at that measurement [32].

**Table 5:** Weights of 13-day-old, western-gray wolf x western-coyote hybrid pups compared with six litters of 14-day-old, western-coyote pups.

	Coyote litters					
Pup	1	2	3	4	5	Hybrid
1	464	562	501	487	582	490
2	467	564	548	564	619	626
3	546	580	555	601	625	636
4	562	587	557	603	646	654
5	598	635	594	646	646	658
6	639	639	601	657	664	720
Ave.(g)	546.0	594.5	559.3	593.0	630.3	630.7
SE	28.6	14.0	14.7	25.3	11.7	31.1

In any case, one of the nine coyote females did produce a normal litter of six live pups and nursed them, demonstrating that gray-wolf semen can inseminate coyote ova, and that a female coyote can carry, deliver, and nurse offspring from the interspecies conception. Whether more than an occasional coyote can do so is an open question, so there might be some evidence from our study that successful hybridization in the wild between male gray wolves and female coyotes, even if they did copulate, might be uncommon.

To definitively determine how likely it is for male gray wolves and western coyotes to hybridize naturally, several more investigations must be done. This study only addresses item 6 above. However, our results do suggest that one of the first steps is to determine whether our hybrids are fertile and to repeat our investigations with the highest quality and quantity of gray-wolf semen and optimizing the timing of inseminations with several more female coyotes. If such studies result in several more failures, that would tend to reduce the need for researching items 1–5.
